# Commentary: The Impact of the Time Interval Between Radiation and Hyperthermia on Clinical Outcome in Patients With Locally Advanced Cervical Cancer

**DOI:** 10.3389/fonc.2019.01387

**Published:** 2019-12-17

**Authors:** Michiel Kroesen, H. Tim Mulder, Gerard C. van Rhoon, Martine Franckena

**Affiliations:** ^1^Department of Radiation Oncology, Erasmus MC, University Medical Center Rotterdam, Rotterdam, Netherlands; ^2^Holland Proton Therapy Center, Delft, Netherlands

**Keywords:** hyperthermia, cervical cancer, thermal dose, clinical outcome, time interval

We thank Crezee et al. for their opinion article concerning the impact of the time interval between radiotherapy (RT) and hyperthermia (HT), elicited by our recent publication showing no effect of the time interval on clinical outcome (1). We welcome the discussion, as time interval between RT and HT is one of the important issues to solve for the hyperthermia community in the near future. As stated in our conclusion, several hyperthermia centers are centralized and receive many patients from radiotherapy departments elsewhere. Also, from a patient perspective, receiving a daily radiotherapy fraction nearby and only traveling to a more distant located hyperthermia facility once a week is preferable and could increase acceptance and tolerance of the thermoradiotherapy treatment.

We regret the feeling of insufficient caution in our conclusions. In fact, we and our referring radiation oncologists took the results of the study of van Leeuwen very seriously. It initiated an intense discussion as to whether the treatment procedure for RT+HT should be offered only if both treatments could be delivered in the same institution to maintain a short treatment interval between the therapies. After all, the international standard for locally advanced cervix cancer consists of combined radiotherapy and platinum-based chemotherapy (2). However, there is a gray zone for elderly patients or patients with larger tumors depending on the confidence in the alternative of combined RT + HT and the regional availability of hyperthermia (3–5). In addition, the adjuvant effect of chemotherapy seems to be less in locally advanced cases and toxicity of thermoradiotherapy is mild (2, 5). We share the common opinion of Crezee et al. that the important clinical consequence of hyperthermia and radiotherapy in the same center should not be based on a single institutions' experience. Hence, our decision to investigate the issue of the time interval in our patient population (6). It was to our honest surprise that we found an absolute null effect of the time interval with Hazard Ratio's of a perfect 1.0 in 400 patients. In our discussion we thought of potential explanations for the different findings between the two studies, including the potential difference in temperatures achieved, as also pointed out by Crezee et al. (7).

A limitation of both our studies, besides the retrospective nature, is the long inclusion period; 1999–2014 for van Leeuwen et al. and 1996–2016 for our cohort. This makes both our analyses subject to confounding factors, because of changes in interval times in different time periods. For example, in our cohort we observed longer waiting times between RT and HT in earlier years vs. more recent years. Given the larger number of patients in our cohort, we were able to analyze the effect of the time interval per time period of 4–5 years, showing no effect of the time interval for all time periods (1).

We agree with Crezee et al. that there is sufficient data showing the effect of hyperthermia on DNA repair inhibition at temperatures higher than 41°C in preclinical models (8–10), but not yet in patients (8). The median temperature increase in our cohort was 40.5°C, which seemed lower than the cohort of van Leeuwen et al. Of note, the difference in temperatures reported in both our cohorts, does not automatically reflect a dose difference to the tumor, as there are distinct differences in the treatment strategies regarding guidance of SAR steering and temperature measurements (11, 12). Looking at our data, however, we observed a wide range in the distribution of TRISE in our cohort, ranging from 0.54 to 5.16°C. We hypothesized that in patients with a high thermal dose, which are also present in our cohort, the effect of the time interval on clinical outcome could be present and could provide an independent confirmation of the finding of van Leeuwen et al. Therefore, we performed an additional analysis for the effect of the time interval on clinical outcome in patients with a high thermal dose. For this, we divided the cohort over the quartiles of TRISE and using univariate Cox proportional hazard analysis, analyzed the effect of the time interval on clinical outcome per TRISE group. As stated by Crezee et al. the medium temperature of their patient group is close to 41°C, which is highly comparable to the temperatures measured in patients of our highest thermal dose group (40.87–42.16°C). Strikingly, also in the group of patients with the highest thermal dose, the Hazard Ratio for Local Control remained 1.0 (95% CI 0.99–1.01; *p* = 0.624) (Table 1). These results strongly indicate that—at least in our cohort—the time interval also has not an effect on outcome in patients with an average target temperature above 40.8°C. Due to the retrospective design of both our studies, without direct comparison with radiotherapy alone, nor with chemoradiotherapy, the effectiveness of hyperthermia cannot be directly proven. However, the strong correlation of thermal dose with clinical outcome in our study is a strong indication of the adjuvant effect of hyperthermia (13).

**Table 1:** Analysis of the effect of the time interval using univariate Cox analysis in patients groups divided over the quartiles of TRISE.

**No of patients**	**TRISE range (^**°**^C)**	**Temperature range (**^°^**C)**	**5-year local control (%)**	**Number of local recurrences**	**HR time interval**	**95% CI of HR**	***p-*value**
94	0.54–2.93	37.54–39.93	60.6	38	1.00	0.99–1.01	0.758
93	2.94–3.46	39.94–40.46	69.3	24	1.00	0.99–1.01	0.769
93	3.47–3.86	40.47–40.86	69.0	24	1.00	0.99–1.01	0.250
93	3.87–5.16	40.87–42.16	72.5	20	1.00	0.99–1.01	0.624

Clearly, the result of our additional analysis for the high thermal dose group will not close the discussion on the relevance of proper selection of the time interval between RT and HT. The results of both cohort studies published on the time interval question, strongly call for further future research in a cooperative manner. We believe, that for the benefit of future patients, further research should be performed at two different levels. First at the clinical level, prospective and uniform measurements and registration on all aspects of hyperthermia treatments, including standardized thermal dose and time interval measurements, should be performed across multiple hyperthermia centers. Second, *in vivo* studies regarding the exact working mechanisms of hyperthermia in patients, not in models, are highly needed. We feel that the Overgaard paper is an important paper, but it represents only a single experimental setup in one tumor model, and can thus not be generalized to the clinic (14).

Finally, fueled by similar level evidence showing conflicting results, the controversy about the effect of the time interval between RT and HT on clinical outcome will be ongoing. We conclude that, during this period, patients with a contraindication or refusing concurrent chemoradiotherapy, can undergo thermoradiotherapy even with long time intervals with the suggestion of no detriment in efficacy compared to short intervals.

## Ethics Statement

The research protocol for this investigation was approved by the medical ethics committee of Erasmus MC Cancer Institute, Rotterdam, the Netherlands (MEC-2018-1081).

## Author Contributions

MK: drafting of the manuscript. HM and MF: critical review of the manuscript. GR: drafting of the manuscript and critical review of the manuscript.

### Conflict of Interest

The authors declare that the research was conducted in the absence of any commercial or financial relationships that could be construed as a potential conflict of interest.
